# Parenting Stress in Autistic and ADHD Children: Implications of Social Support and Child Characteristics

**DOI:** 10.1007/s10803-024-06377-4

**Published:** 2024-05-03

**Authors:** Ana Pardo-Salamanca, Eva Rosa-Martínez, Soledad Gómez, Cristina Santamarina-Siurana, Carmen Berenguer

**Affiliations:** 1 Department of Developmental and Educational Psychology, Faculty of Psychology and Speech Therapy, University of Valencia - Spain, Avda. Blasco Ibáñez, 21, Valencia, 46010 Spain; 2 Department of Basic Psychology- ERI-Lectura, University of Valencia, Valencia, Spain; 3 Catholic University of Valencia, Campus Capacitas, Valencia, 46010 Spain; 4 Polytechnic University of Valencia, Valencia, Spain; 5 Department of Developmental and Educational Psychology-ERI-Lectura, Faculty of Psychology and Speech Therapy, University of Valencia, Avenida Blasco Ibáñez, 21, Valencia, 46010 Spain

**Keywords:** Parenting stress, ASD, ADHD, Social support, Child characteristics

## Abstract

High levels of parenting stress have been detected in mothers of children with Autism (ASD) and children with attention deficit hyperactivity disorder (ADHD) comparing with mothers of typically developing (TD) children. The current study explored the implications of social support (confidant and affective support) and child characteristics (emotional, behavioral and sleep problems) on parenting stress in ASD and ADHD. Furthermore, the differences between mothers of children with autism, ADHD and TD on the studied variables were examined.A total of 120 mothers of 30 TD children, 47 with ASD without intellectual disability and 43 with ADHD collaborated in the study. Significant differences were found between clinical and TD groups in parenting stress, social support, and child characteristics.Correlation analysis in the group with ADHD revealed that parental stress correlated significantly with social support and with children’s emotional problems. In the ASD group, parental stress also correlated significantly with children’s sleep and behavioral problems. Moreover, multiple regressions showed that confidant support was a significant predictor of parenting stress in both clinical groups.The findings provide new insights to consider social support as a fundamental part of treatments for parenting stress in mothers of children with ASD and ADHD.

Parenting is intrinsically rewarding but also presents myriad challenges that can be stressful for many parents (Deater-Deckard, 2004). For parents of children with developmental disorders like Autism Spectrum Disorder (ASD) or Attention Deficit Hyperactivity Disorder (ADHD), these challenges can be intensified. ASD is characterized by social interaction and communication difficulties, as well as restrictive, repetitive, and stereotypical behaviors (American Psychiatric Association, 2013). In contrast, ADHD involves symptoms of inattention and/or hyperactivity/impulsivity that cause great impairments in different domains of adaptive functioning in daily life (APA 2013). Moreover, both conditions present comorbid problems such as social and behavioral difficulties (Gargaro et al., 2011), and daily life dysfunctions, such as sleep problems (Martin et al., 2019), which entails parenting stress, and low social support (Riany & Ihsana, 2021; Robinson & Weiss, 2020).

Parenting stress is a multidimensional concept that arises when parents’ perceptions of parenting demands and the availability of resources to address those demands are imbalanced (Deater-Deckard, 2004). From this multidimensional theoretical framework, determinants of parental stress related to the characteristics of the child, parents, family and environment have been identified (Abidin, 1995).

Previous studies have indicated that parents of children with ASD or ADHD often experience higher levels of stress compared to parents of typically developing (TD) children, and that the relationship between parental stress and neurodevelopmental disorders is bidirectional (Berenguer et al., 2021; Hutchison et al., 2016; Iwamoto et al., 2023; Theule et al., 2013). Parenting stress has been associated with increased behavioral problems, internalizing and externalizing symptoms, and adaptive problems such as sleep disturbances in children (Deater-Deckard, 2004; Whelan et al., 2022). Specifically, several studies have demonstrated that children’s emotional and behavioral difficulties predict higher levels of parenting stress over time (Baker et al., 2023). Moreover, children’s behavioral problems also negatively impact the quality of parent-child interactions (Stephenson et al., 2022). Thus, parents who report higher levels of parenting stress also typically experience less pleasure and increased negative interactions with their children (Abidin, 1995).

In particular, raising a child with ASD raises specific challenges that may contribute to maternal distress, related to the management of the child’s symptomatology and behavioral characteristics, which also depends on the characteristics of the parents themselves, including their perception of the child behavior problems and social support (Miranda et al., 2019; Zaidman-Zait et al., 2017; Weinberg et al., 2021). Mothers of children with autism are more likely to experience less social support when compared to mothers of children without autism (Enea & Rusu, 2020). The experience of low social support may also be affected by child characteristics, such as emotional, behavioral, and sleep problems (Iwamoto et al., 2023; Mannion & Leader, 2023). Previous research has found that sleep and behavioral problems in children with autism are related to increased levels of stress and less social support in their mothers (Mannion & Leader, 2023).

Regarding ADHD, it is well established that the frequency of ADHD symptoms in children is associated with significantly greater global parenting stress (Galloway et al., 2019; Theule et al., 2013). Specifically, research has reported that children’s emotional, behavioral and sleep problems predict parenting stress (Martin et al., 2019; Flynn et al., 2021). Unsurprisingly, parenting a child with ADHD is linked to increased stress and conflict within the family, decreased child and parental quality of life (Galloway et al., 2019), and lower levels of perceived social support (Riany & Ihsana, 2021). Overall, ADHD in children places greater demands on parents to manage child behavior, while also requiring greater resources from the parent to address their child’s difficulties.

Few studies have examined stress levels between families of children with ASD without intellectual disability and children with ADHD, with inconsistent findings. Schiltz et al. (2022) evidenced that parents of children with autism and ADHD comorbidity reported greater parenting stress compared to parents of children with autism alone, while Hutchison and colleagues (2016) did not detect differences in parenting stress between families of children with autism and children with ADHD. These discrepant findings may be related to potential differences in measurement tools or sample characteristics (Schiltz et al., 2022).

It is essential to improve our understanding of the factors that predict parental stress because of the great impact it has on mothers’ health, and because increased levels of stress in mothers of children with ASD and ADHD could affect child developmental outcomes and perceived social support (Cochrane et al., 2023; Craig et al., 2016). Social support is one of the most powerful predictors of psychological adjustment among parents raising children with ASD and other disabilities. Furthermore, social support has been identified as a critical factor that has been linked to improved mental health in mothers of children with autism and mothers of children with ADHD (Schiltz et al., 2022; Theule et al., 2013). In particular, the quality of the informal support, such as that provided by friends and family, has been shown to be effective in reducing stress among mothers of children with autism (Benson, 2012; Drogomyretska et al., 2020).

The parental characteristics used for predicting parenting stress among families of children with autism and children with ADHD in previous studies mainly focused on parents’ psychopathology and parental clinical symptoms (Craig et al., 2016; Theule et al., 2013). However, the relationship between social support and parenting stress in children with autism and children with ADHD has been insufficiently studied.

Therefore, this study aimed to explore the implications of social support (confidant support and affective support) and child characteristics (emotional/behavioral and sleep problems) on parenting stress in mothers of children with ASD without intellectual disability and children with ADHD. Furthermore, the differences between autism, ADHD, and TD on parenting stress, social support, and child characteristics were examined.

We predicted that parenting stress in mothers of children with ASD or ADHD would be elevated relative to a TD comparison group. We hypothesized that perceived poor social support and children’s emotional/behavioral problems and sleep disturbances, would be associated with high rates of parental stress in mothers of children with ASD without intellectual disability and children with ADHD.

## Methods

### Participants

The sample of this study was formed by 120 mothers of 30 TD children, 47 with ASD without intellectual disability and 43 with ADHD (matched on age, and IQ) (see Table 1). To reduce variability, only mothers were included in the present study given gender-based differences in parenting stress (Schiltz et al., 2022). A majority of the sample were mothers who were married or living in common-law relationships (*n* = 92; 76.6% (ASD group = 34, ADHD group = 30, TD group = 28), with 2 identifying as single (1.6%; 1 in ADHD group and 1 in TD group) and another 26 (21.6%) identifying as separated or divorced (ASD group = 13, ADHD group = 12, TD group = 1). In relation to maternal employment as indicated in Tables 1, 52 mothers had skilled jobs (jobs that require specialized studies), 41 had unskilled jobs and 27 mothers were unemployed. Considering the number of working hours per week, most mothers were working between 25 and 35 h per week, in all groups.

Children ranged in age from 8 to 12 years (M = 8.40; SD = 1.62), while mothers ranged in age from 35 to 55 years (M = 42.64; SD = 5.00). There were no significant differences in mothers age across groups, *F*(2, 116) = 2.61 *p* = .073.

The children were recruited from specialized psychoeducational centers and clinical health centers in Spain. To be considered eligible, children must have been formally diagnosed with ASD without intellectual disability or ADHD by a qualified psychologist or medical doctor. ADHD children meet the clinical diagnostic criteria with combined presentation based on the Diagnostic and Statistical Manual of Mental Disorders Fifth Edition, DSM-5 (APA, 2013). Parents and teachers filled out the 18 diagnostic criteria from the DSM-5 (APA, 2013), indicating the severity of each item from 0 to 3. The requirements were the presence of at least six inattention symptoms and/or six other hyperactivity/impulsivity symptoms. Table 1 shows the raw score of the parents’ version DSM-5 questionnaire in both clinical groups ASD and ADHD).

ASD children meet the criteria of Autistic Diagnostic Interview—Revised (ADI-R; Rutter at al. 2006), and/or the Autism Diagnostic Observation Schedule, Generic (ADOS G; Lord et al., 2000). The Diagnostic and Statistical Manual of Mental Disorders- Fifth Edition, DSM-5, (APA 2013) was used to confirm current symptom severity. Additionally, to confirm the ASD diagnosis in both clinical groups, a trained psychologist administered the Social Communication Questionnaire (SCQ; Rutter et al., 2003). None of the participants with autism had been formally diagnosed with ASD + ADHD. For all groups the composite IQ score of ≥ 80 on the Kaufman Brief Intelligence Test, 2nd Edition (KBIT-2; Kaufman & Kaufman, 2004) was required. The prevalence of sleep problems among children was TD (*n* = 7, 23.3%), ASD (*n* = 32, 68,1%) and ADHD group (*n* = 33, 76,7%). Moreover, 34 (79%) children in the ADHD sample were receiving psychostimulant medication and 19 (40.4%) children in the ASD group were receiving antipsychotic medications (mostly risperidone) for behavioral problems. None of the clinical groups were receiving sleep medication at the time of the evaluation. In relation of the number of siblings, 47.5% (*n* = 57) children had no siblings (ASD group = 22, ADHD group = 23, TD group = 12), 51.6% (*n* = 62) children had from 1 to 3 siblings (ASD group = 25, ADHD group = 20, TD group = 17), and 1 child with TD had 4 siblings. Only four children with ASD had one brother with the same clinical condition, according to information reported by parents.

Children with TD were selected from public primary schools. None of them presented a history of psychopathologies or criteria for ASD or ADHD diagnosis according to DSM-5. The exclusion criteria for all groups were having an IQ of less than 80, motor or sensorial deficits, mental or neurological diseases.

**Table 1:** Sociodemographic characteristics

	ASD (*n* = 47)		ADHD (*n* = 43)		TD (*n* = 30)		Statistics	
*Child characteristics*	M	SD	M	SD	M	SD	F _(2,116)_	*p*
Age	9.37	1.75	9.29	1.38	8.66	1.32	1.69	0.171
IQ	98.37	10.92	98.94	6.91	100.02	8.21	0.48	0.611
Sex (% boys)	89.3%		90.7%		65.6%			
ASD Symptoms (ADI-R)	28.3	5.7	-	-	-	-		
ASD Symptoms (SCQ)	23.5	7.0	10.8	6.2	-	-		
ADHD Symptoms (DSM-5)	24.6	8.7	41.0	9.1	-	-		
Medication (% yes)	40.4	-	79.0%	-	0.0%	-		
Number of siblings	0.98	0.86	0.85	0.61	1.12	0.72		
*Mother characteristics*	N	%	N	%	N	%	F/χ^2^	*p*
Maternal education							0.57	0.564
Highschool or Higher Education (%yes)	20	42.6	19	44.2	17	56.6		
Maternal employment (%)							0.93	0.924
Skilled job	20	42.5	18	41.9	14	46.6		
Unskilled job	18	38.3	12	27.9	11	36.6		
Unemployed	9	19.1	13	30.2	5	16.6		
Working hours/week (%)								
Skilled job < 25 h	5	25	2	11.1	2	14.2		
Skilled job 25–35 h	13	65	15	83.3	8	57.1		
Skilled job > 35 h	2	2	1	5.5	4	28.5		
Unskilled job < 25 h	4	22.2	4	33.3	2	18.2		
Unskilled job < 25–35	12	66.6	7	58.3	7	63.6		
Unskilled job > 35 h	2	11.1	1	8.3	2	18.2		

DSM-5 parents’ version (Diagnostic and Statistical Manual of Mental Disorders- Fifth Edition), SCQ total score (Social Communication Questionnaire), ADI-R total score (Autistic Diagnostic Interview—Revised), h (hours per week)

* p < .05

### Measures

*Parenting Stress Index – Short Form* (PSI-SF; Abidin, 1995; adapted to Spanish by Díaz-Herrero et al., 2011). The PSI-SF is a self-report questionnaire of 36 items that measures total parenting stress. The 36 items are distributed in three subscales of 12 items each (parental distress, parent–child dysfunctional interaction, and difficult child) rated on a five-point Likert-type response scale. The scale provides a measure of total stress by adding up the scores on the 36 items, with a total score above 90 being clinically significant. The total score of PSI-SF completed by the mothers was used in this study. The PSI-SF presents good psychometric properties, and it is an effective measure for use with high-risk families (Barroso et al., 2016). The internal consistency for the total scale was high in our sample (Cronbach’s *α* = 0.92).

*Strengths and Difficulties Questionnaire* (SDQ; Goodman, 1997; Spanish adaptation by Rodríguez-Hernández et al., 2012). The SDQ can be filled out by parents or teachers to rate a wide range of psychopathological symptoms and prosocial behavior in children and adolescents between 4 and 16 years old. The questionnaire consists of five subscales (emotional symptoms, hyperactivity/inattention, conduct problems, peer problems and prosocial behaviour). A total difficulties score (SDQ total) comprising the first four subscale scores indicate the overall extent of a child’s psychopathological problems. Each of the five scales incorporates 3 response alternatives (not true, somewhat true, very true). Higher scores indicate more difficulties. For this study, mothers reported about the emotional and behavioral difficulties subscales. The SDQ presents adequate psychometric properties with adequate reliability (between 0.73 and 0.76) (Rodríguez-Hernández et al., 2012). The internal consistency (Cronbacht’s a) of the emotional and behavioral difficulties subscales for our sample was 0.76 and 0.77, respectively (Goodman, 1997).

*The Sleep Disturbance Scale for Children* (SDSC; Bruni et al., 1996; Spanish adaptation by Pagerols et al., 2023) is a 26-item parent-reported questionnaire that assesses the occurrence of sleep disturbances during the last 6 months in children aged 6–16 years. Mothers rated their children sleep behaviour on a 5-point Likert scale (never to always). This instrument differentiates between conditions such as disorders in initiating and maintaining sleep, respiratory disorders, sleep arousal, sleep-wake transition disorders, excessive sleepiness, restless legs syndrome/periodic limb movement syndrome/growth pains, and sleep hyperhidrosis. The total sleep problems score was obtained by adding all subscale scores and was used in the present study. The internal consistency was α = 0.79 and test–retest reliability *r* = .71 over a 6-month interval (Bruni 1996).

*Social Functional Support Questionnaire Duke-UNC* (Broadhead et al., 1988; Spanish adaptation; Bellón et al. 1996). This questionnaire was filled out by mothers and measures the subjects’ perception of the availability of help from family and friends in difficult situations, as well as the ease of communication with them. The instrument comprises 11 questions with a Likert-type scale ranging from 1 = “much less than I would like” to 5 = “as much as I would like”. The questionnaire assesses two factors: confidant support (ability to rely on individuals for communication) and affective support (demonstrations of love, affection, and empathy). Confidant support scale has a total of six items and affective social support includes five items. The present study used the direct scores on both scales. Consistent with the results of the Spanish adaptation (Bellón et al. 1996), the internal consistency (Cronbacht’s a) of the confidant support and affective scales for our sample was 0.85 and 0.76, respectively.

### Procedure

Ethical approval was granted by the Universitat de València Human Research Ethics Committee (UV-INV_ETICA-1,905,517). The parents provided their written informed consent to collaborate in this study. Mothers completed the questionnaires in one session with the research team that had been trained in the administration of the questionnaires used.

## Data Analysis

Data were first screened for normality and univariate and multivariate outliers. Missing data at the item level were handled. Also differences between groups on demographic variables and autism symptoms to identify potential covariates. To address the first aim, statistical analysis of data was performed using the SPSS Statistical Software Version 26. Multivariate Analyses of Variance (MANOVA) were conducted to examine group differences (ASD without intellectual disability, ADHD and TD) in parenting stress total index (PSI), confidant support, affective support, SDQ-variables and sleep problems. A p-value significance level cut-off of < 0.008 was used applying Bonferroni correction for multiple comparison, to reject the null hypothesis for the analyses. Moreover, we conducted pairwise comparisons using Tukey’s post hoc analyses. To address the second aim, bivariate Pearson correlations were performed to test relationships between parenting stress, social support, behavioral/emotional difficulties, and sleep problems among clinical groups (ASD and ADHD). Following, multiple linear regression analysis was used to whether the children’s characteristics and parents perceived social support predicted the criterion parental stress, in ASD and ADHD groups. Specifically, univariate model was built with the predictors (social support variables, SDQ measures and sleep problems) for each group. Also, demographic variables (age, sex, IQ) were evaluated and subtracted from the outcome variable (parenting stress). The variables associated with significant changes in the univariate analysis (*p* < .05) and the clinically relevant variables were included in a multiple linear regression model. For correlation and multiple linear regression, statistical analyses were conducted in RStudio version 1.3.1073 (RStudio Team, 2020).

## Results

### Differences Between ASD, ADHD and TD Groups

The first aim of this study was to explore the differences between the groups of mothers of children with ASD, ADHD and TD on parenting stress, perceived social support and child’ emotional/behavioral and sleep problems. The MANOVA performed to evaluate the main effect of group on parenting stress, social support, SDQ variables and sleep problems was statistically significant [Wilk‘s Lambda (Λ) = 0.17, F(16,220) = 19.02, *p* < .001, η2p = 0.58]. At the univariate level, a significant group effect emerged for all outcome variables with the Bonferroni correction (see Table 2). In terms of parenting stress, Tukey’s post hoc tests revealed that the TD group exhibited significantly lower parenting stress than the ASD and ADHD groups (*p* < .01) while the ADHD group did not significant differ from ASD group (*p* > .05). Regarding social support variables (confidant support and affective support) results evidenced statistically significant differences between clinical groups and TD group without significant differences between clinical groups. A similar pattern emerged in terms of sleep problems, and SDQ variables (emotional and behavioral difficulties) such that Tukey’s post hoc tests revealed that the children with ASD and ADHD exhibited significantly more children sleep problems and more emotional/ behavioral difficulties than children with TD (*p* < .01) while the ADHD group did not significant differ from ASD group (*p* > .05).

**Table 2:** Differences between ASD, ADHD and TD groups on variables of the study

	ASD (*n* = 47)		ADHD (*n* = 43)		TD (*n* = 30)				
	M	SD	M	SD	M	SD	F _(2,116)_	η^2^_P_	Group differences
PSI	121.75	20.8	113.21	22.91	64.90	11.9	85.38*	0.59	ASD, ADHD > TD
Support c	15.82	6.32	16.64	7.60	23.59	4.12	16.06*	0.21	ASD, ADHD > TD
Support a	12.44	5.08	14.10	5.72	19.38	3.13	19.56*	0.25	ASD, ADHD > TD
SDQ-E	6.06	2.11	6.05	2.43	1.59	1.01	64.02*	0.52	ASD, ADHD > TD
SDQ-B	4.91	2.18	4.85	1.97	1.35	1.36	39.22*	0.40	ASD, ADHD > TD
Sleep p	58.88	20.31	57.23	20.46	35.68	5.13	18.93*	0.24	ASD, ADHD > TD

PSI (total parenting stress index), Support c (confidant support), Support a (affective support), SDQ-E (SDQ emotional problems), SDQ-B (behavioral problems), Sleep p (total sleep problems)

p* < 0.008 (Bonferroni correction for multiple comparison)

### Implication of Social Support and Child Characteristics on Parenting Stress in ASD and ADHD Groups

**Fig. 1:**
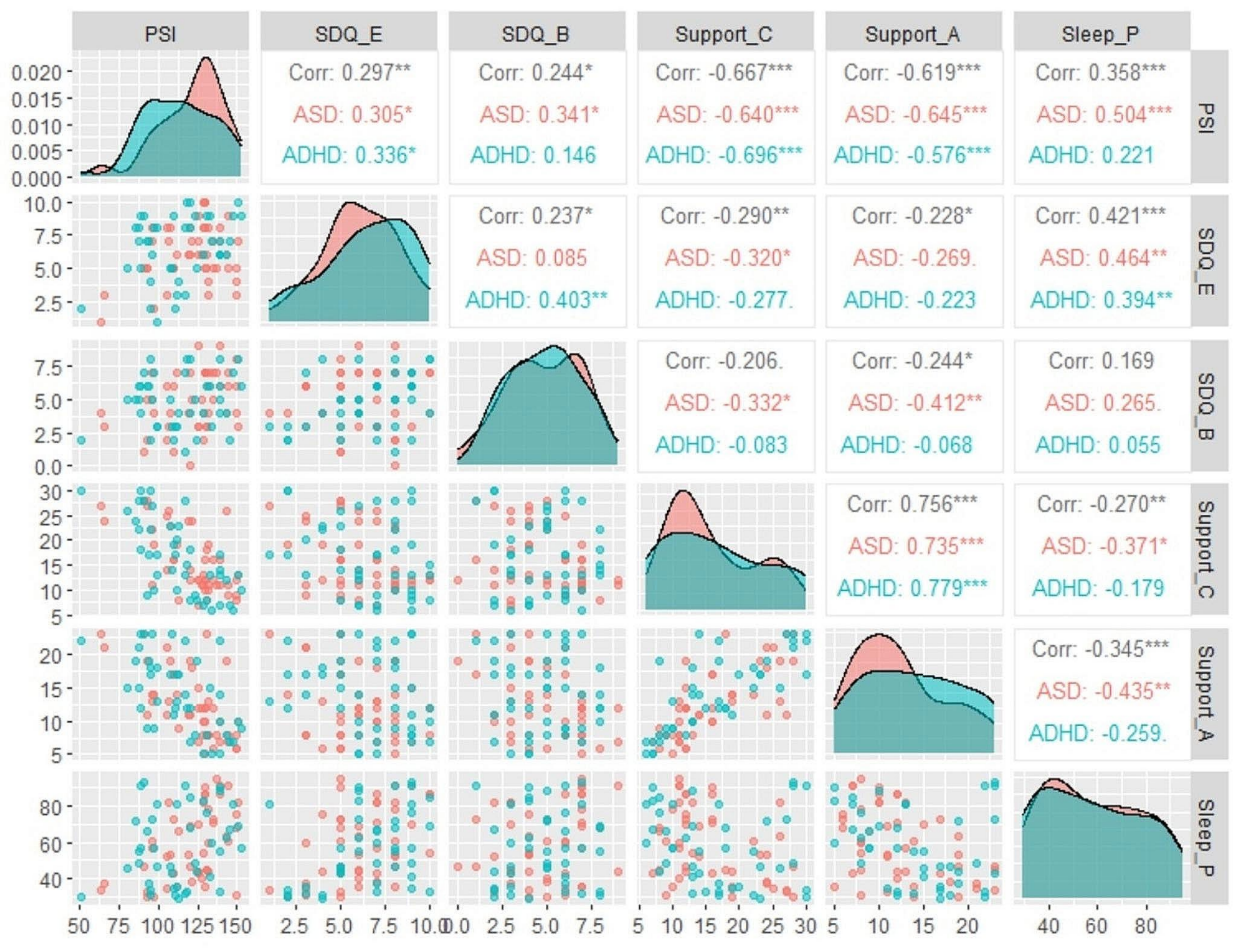
Correlation matrix among mothers and children variables in ASD and ADHD groups. PSI (total parenting stress index), Support c (confidant support), Support a (affective support), SDQ-E (SDQ emotional problems), SDQ-B (behavioral problems), Sleep p (total sleep problems) *p < .05

Bivariate Pearson correlations by groups (ASD and ADHD) were applied to further explore the association of parental stress total index with confidant support, affective support, SDQ-emotional problems, SDQ-behavioral problems, and sleep problems. Results showed statistically significant associations in the correlation analysis (Fig. 1).

According to the matrix, parenting stress in ASD group, had a negative correlation with confidant and affective support (*p* > .001). On the contrary, a positive significant association were observed between parenting stress and children sleep problems (*p* > .001) and children emotional/behavioral difficulties (*p* = .02). Similarly, in ADHD group, parenting stress had a negative significant association with confidant and affective support (*p* > .001). Besides, parenting stress was positively correlated with emotional difficulties (*r* = .33), with a P value of 0.020. We did not observe any statistically significant correlation between parenting stress and behavioral difficulties (*p* = .35) and between parenting stress and sleep problems (*p* = .15) among ADHD group.

Multiple linear regression analysis was conducted in the group of ASD with the studied variables, resulting as follows: SDQ Emotional problems (β = -0.16, 95% CI [-0.49, 0.17], *p* = .326), SDQ Behavioral problems (β = 0.17, 95% CI [-0.14, 0.49], *p* = .264), Confidant support (β = -0.78, 95% CI [-1.17, -0.40], *p* < .000), Affective support (β = 0.01, 95% CI [-0.39, 0.41], *p* = .949) and Sleep problems (β = 0.23, 95% CI [-0.11, 0.58], *p* = .178). The percentage of variance explained by the model was 61% (Table 3).

**Table 3:** Multiple linear regression results using parenting stress as the criterion in ASD group

Predictor	b	B 95% CI [LL, UL]	beta	Beta 95% CI [LL, UL]	sr^2^	sr^2^ 95% CI [LL, UL]	*r*	Fit
(Intercept)	42.83*	[5.75, 79.92]						
SDQ_E	-1.48	[-4.51, 1.55]	-0.16	[-0.49, 0.17]	0.01	[-0.03, 0.06]	0.30	
SDQ_B	1.46	[-1.16, 4.08]	0.17	[-0.14, 0.49]	0.02	[-0.04, 0.07]	0.42*	
Support c	-2.53**	[-3.77, -1.28]	-0.78	[-1.17, -0.40]	0.22	[0.03, 0.41]	− 0.75**	
Support a	0.05	[-1.48, 1.58]	0.01	[-0.39, 0.41]	0.00	[-0.00, 0.00]	− 0.67**	
Sleep p	0.22	[-0.10, 0.54]	0.23	[-0.11, 0.58]	0.02	[-0.04, 0.09]	0.49**	
								*R*^*2*^ = 0.616**
								95% CI[0.29,0.71]

Support c (confidant support), Support a (affective support), SDQ-E (SDQ emotional problems), SDQ-B (behavioral problems), Sleep p (total sleep problems).

* *p* < .05

** *p* < .01

Multiple linear regression analysis was conducted in the group of ADHD, with the studied variables, resulting as follows: SDQ Emotional problems (β = 0.04, 95% CI [-0.29, 0.37], *p* = .793), Confidant support (β = -0.55, 95% CI [-1.05, -0.04], *p* = .036), and Affective support (β = -0.32, 95% CI [-0.82, 0.19], *p* = .209). The percentage of variance explained by the model was 49% (Table 4).

**Table 4:** Multiple linear regression results using parenting stress as the criterion in ADHD group

Predictor	b	B 95% CI [LL, UL]	beta	Beta 95% CI [LL, UL]	sr^2^	sr^2^ 95% CI [LL, UL]	*r*	Fit
(Intercept)	43.98**	[14.99, 72.98]						
SDQ_E	0.39	[-2.59, 3.36]	0.04	[-0.29, 0.37]	0.00	[-0.02, 0.02]	0.22	
Support c	-1.55*	[-2.99, -0.10]	-0.55	[-1.05, -0.04]	0.09	[-0.06, 0.23]	− 0.75**	
Support a	-1.20	[-3.12, 0.71]	-0.32	[-0.82, 0.19]	0.03	[-0.05, 0.11]	− 0.67**	
offset(Sex + Age + IQ)	NA	[NA, NA]	NA	[NA, NA]	NA	[NA, NA]	− 0.46**	
								*R*^*2*^ = 0.487**
								95% CI[0.16,0.63]

Support c (confidant support), Support a (affective support), SDQ-E (SDQ emotional problems)

* *p* < .05. ** *p* < .01

## Discussion

The goals of this study were to examine the differences regarding parenting stress, social support, and child characteristics (emotional/behavioral and sleep problems) from the perceptions of mothers of children with autism without intellectual disability, children with ADHD, and typically developing children. Secondly, the current study aimed to elucidate the roll of perceived social support by mothers and child characteristics (emotional/behavioral and sleep problems) on parenting stress in the two clinical groups (ASD and ADHD).

Regarding the first hypotheses, the results confirmed that mothers of children with autism without intellectual disability and children with ADHD, compared to mothers of children with TD, present significantly higher levels of parenting stress, lower perception of social support, and perceive more emotional, behavioral, and sleep problems in their children. More specifically, the comparison of parenting stress, perception of social support, and child emotional, behavioral, and sleep problems revealed non-significant differences between the clinical groups. These results are linked with those obtained among parents of children with autism and children with ADHD (Arias-Mera et al., 2023; Craig et al., 2016; Hutchison et al., 2016; Riany & Ihsana, 2021). The above findings may stem from the similarities in the social and behavioral functioning of ASD without disability and ADHD (Gargaro et al., 2011), in addition to a specific pattern of functioning in families of children with ASD and/or ADHD, who face more challenges in parenting than families with TD children (Arias-Mera et al., 2023; Mannion & Leader, 2023).

As for the second hypothesis, it was partially confirmed the association of parenting stress with social support, children’s emotional/behavioral, and sleep problems. Consistent with previous studies, perceived social support, in both dimensions, and child emotional difficulties were highly correlated with parental stress among mothers of children with autism and children with ADHD (Linsey & Barry, 2018; Martin et al., 2019; Schiltz et al., 2022; Weinberg et al., 2021). However, only in the ASD group were statistically significant associations found between parenting stress and behavioral difficulties and sleep problems. Thus, the mothers of children with autism without intellectual disabilities, encountering difficult situations related to child behavior and sleep problems, experienced a higher level of stress, as indicated by previous research (Mannion & Leader, 2023). Surprisingly, we did not find a significant association between parenting stress and behavioral and sleep problems in the ADHD group probably due to the small sample size. Since the results of previous studies among mothers of children with ADHD are also mixed in this sense future research should examine this further (Martin et al., 2019; Theule et al., 2013).

Moreover, this study evidenced that social support, specifically confidant support, was the only significant predictor for parenting stress in mothers of children with autism and children with ADHD. Confidant support was characterized in terms of the perceived availability of family members and friends for communication and obtaining emotional support (Benson, 2012). Based on our findings, it seems that the possibility of having friends or family to communicate with enables mothers to face many challenges and demands associated with parenting a child with ASD or ADHD without experiencing high levels of stress. In contrast, a lack of social support, in particular confidant support, may lead to a high level of parenting stress, based on prior research (Craig et al., 2016; Riany & Ihsana, 2021).

These findings are important since it could be a significant element in building effective support programs for families of children with ASD without intellectual disabilities and children with ADHD.

The current study adds to the literature by elucidating the differential contributions of social support, behavioral, emotional difficulties, and sleep problems to parenting stress in mothers of children with ASD and children with ADHD. Examining protective factors such as social support with child problems in both neurodevelopmental disorders, rather than exploring each disorder separately, adds value to the current study. First, because both disorders share common characteristics and secondly, this study highlights the importance of social support as a key factor associated with reduced parental stress. To our knowledge, this is the first study that addresses the implication of social support and child emotional, behavioral and sleep problems to parental stress of mothers of children with ASD without intellectual disabilities and children with ADHD.

Finally, it is important to consider some limitations in the present study. First, the sample size is not very large, which affects the generality of the findings. Also, this is a cross-sectional study, examining parenting stress at only one point in time, so it does not consider the potential developmental changes in parenting stress across time. Therefore, longitudinal studies are needed. Additionally, the IQ range of the sample is also limited to those without intellectual disability, and therefore, results may not be representative of children with autism and lower IQ. Moreover, the measures here were limited to mother report, and thus, due to potential bias from shared methods variance, additional information from other informants, such as fathers, and/or third-party observers like teachers, would enrich these findings. Lastly, the clinical groups did not present clinical comorbidities between ASD and ADHD. It would be interesting to examine stress in parents of children with both ASD + ADHD co-ocurring condicions compared to parents of children with ASD or ADHD only. Also, our sample was composed mainly by co-parents. Future research could examine the differences in levels of stress in single parents compared to co-parents in children with ASD + ADHD.

Through this study, we hope to enhance our understanding of the multifaceted challenges faced by mothers of children with ASD and ADHD. By spotlighting the critical factors that influence parenting stress, we can pave the way for more effective, tailored interventions that address the needs of mothers of children with ASD and ADHD and strengthen their resilience. Additionally, this study clarifies the effect of social support on parenting stress in mothers of children with autism without intellectual disability and children with ADHD. Future research should continue examining the contribution of protective factors such as social support or coping abilities with child characteristics in more varied and larger samples to further contribute to understanding the development of parenting stress in families of children with neurodevelopmental disorders. Other studies should analyze the contribution of parental social-demographic factors such as marital status, number of working hours or number of siblings on parenting stress and its effects in children with autism or ADHD.
